# μ_2_-Acetato-κ^2^ *O*:*O*′-(4,4′-bipyridyl-κ*N*)tris­(diethyl di­thio­phosphato-κ^2^ *S*,*S*′)-μ_3_-sulfido-tri-μ_2_-sulfido-trimolybdenum(IV) diethyl ether monosolvate

**DOI:** 10.1107/S1600536814012501

**Published:** 2014-06-04

**Authors:** Keisuke Kawamoto, Isamu Kinoshita

**Affiliations:** a Department of Chemistry, Graduate School of Science, Osaka City University, Sumiyoshi-Ku, Osaka 558-8585, Japan

## Abstract

In the title compound, [Mo_3_(CH_3_COO)(C_4_H_10_O_2_PS_2_)_3_S_4_(C_10_H_8_N_2_)]·C_4_H_10_O, the complex mol­ecule has a trinuclear incomplete cuboidal structure which is coordinated by three kinds of ligands, namely, diethyl di­thio­phosphate, acetate and 4,4′-bipyridyl. If Mo—Mo bonds are ignored, each Mo atom can be considered as six-coordinated in a distorted octa­hedral geometry. The Mo—Mo distance of 2.6880 (5) Å for two the Mo atoms bridged by the acetate ligand is shorter than the other two Mo—Mo distances [2.7490 (5) and 2.7566 (5) Å]. One ethyl group is disordered between two conformations in a 0.65 (3):0.35 (3) ratio. In the crystal, weak C—H⋯O inter­actions link the trinuclear mol­ecules related by translation in [100] into chains. The crystal packing exhibits short inter­molecular S⋯S contacts of 3.1886 (13) Å. In other words, in this crystal packing, a supramolecular structure is constructed by the C—H⋯O and S⋯S interactions.

## Related literature   

For related compounds and their crystal structures, see: Hernandez-Molina *et al.* (2011 ▶); Ogino *et al.* (1998 ▶); Tang *et al.* (2001 ▶); Yao *et al.* (1995 ▶).
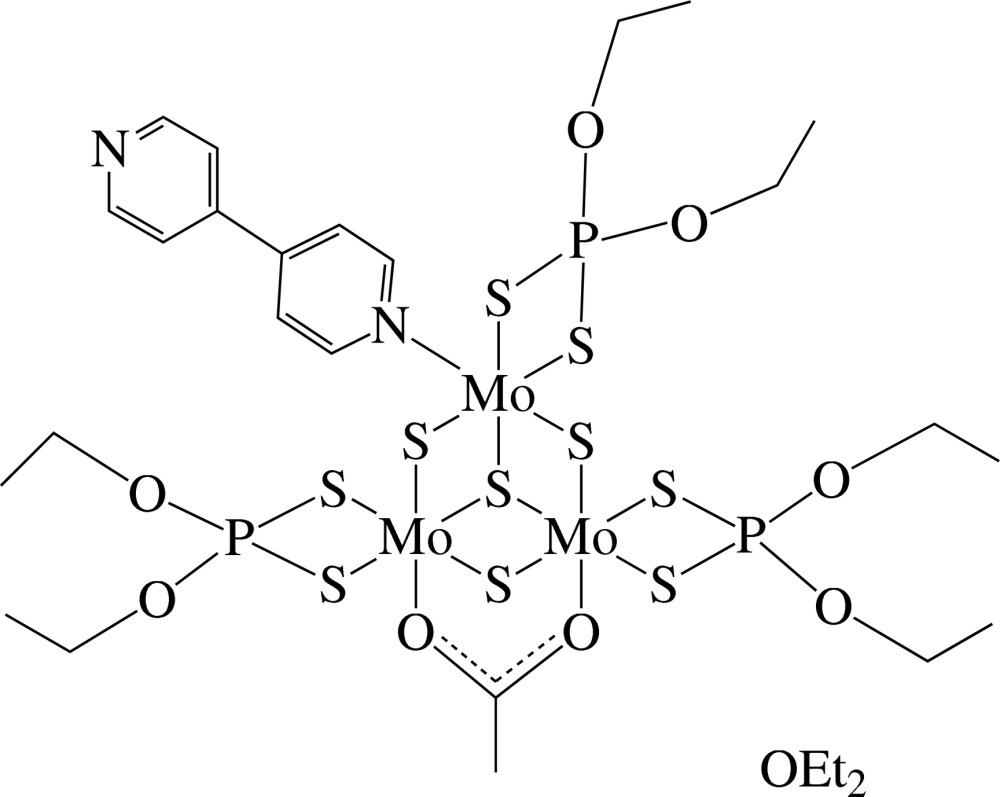


## Experimental   

### 

#### Crystal data  

[Mo_3_(C_2_H_3_O_2_)(C_4_H_10_O_2_PS_2_)_3_S_4_(C_10_H_8_N_2_)]·C_4_H_10_O
*M* *_r_* = 1261.06
Monoclinic, 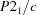

*a* = 13.1573 (16) Å
*b* = 27.647 (3) Å
*c* = 14.2153 (17) Å
β = 107.7183 (16)°
*V* = 4925.6 (10) Å^3^
*Z* = 4
Mo *K*α radiation
μ = 1.32 mm^−1^
*T* = 153 K
0.19 × 0.16 × 0.03 mm

#### Data collection  

Rigaku Saturn724 diffractometer
Absorption correction: multi-scan (*REQAB*; Rigaku, 1998 ▶) *T*_min_ = 0.778, *T*_max_ = 0.961
40597 measured reflections
11260 independent reflections
9267 reflections with *F* ^2^ > 2σ(*F* ^2^)
*R*_int_ = 0.041

#### Refinement  

*R*[*F* ^2^ > 2σ(*F* ^2^)] = 0.040
*wR*(*F* ^2^) = 0.095
*S* = 1.08
11260 reflections
506 parameters
1 restraint
H-atom parameters constrained
Δρ_max_ = 0.78 e Å^−3^
Δρ_min_ = −0.73 e Å^−3^

### 

Data collection: *CrystalClear* (Rigaku, 2010 ▶); cell refinement: *CrystalClear*; data reduction: *CrystalClear*; program(s) used to solve structure: *SIR92* (Altomare *et al.*, 1994 ▶); program(s) used to refine structure: *SHELXL97* (Sheldrick, 2008 ▶); molecular graphics: *CrystalStructure* (Rigaku, 2010 ▶); software used to prepare material for publication: *CrystalStructure*.

## Supplementary Material

Crystal structure: contains datablock(s) General, I. DOI: 10.1107/S1600536814012501/cv5459sup1.cif

Structure factors: contains datablock(s) I. DOI: 10.1107/S1600536814012501/cv5459Isup2.hkl

CCDC reference: 1005735

Additional supporting information:  crystallographic information; 3D view; checkCIF report

## Figures and Tables

**Table 1:** Hydrogen-bond geometry (Å, °)

*D*—H⋯*A*	*D*—H	H⋯*A*	*D*⋯*A*	*D*—H⋯*A*
C11—H11*B*⋯O5^i^	0.99	2.38	3.335 (7)	161

Symmetry code: (i) 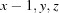.

**Fig. 1.**
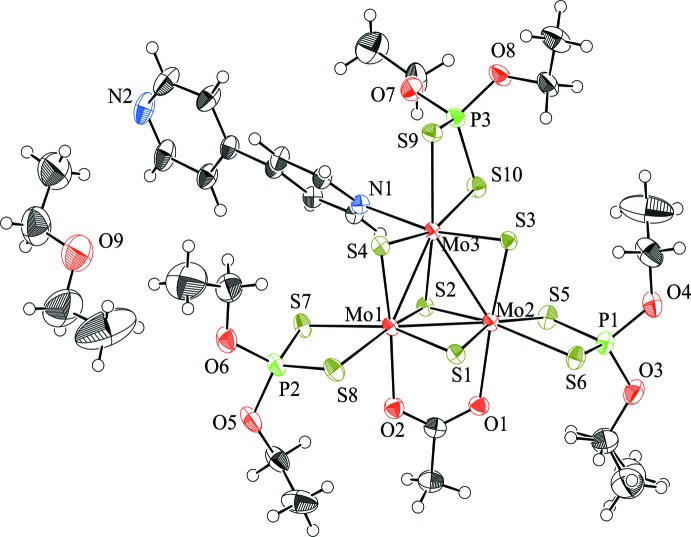
The molecular structure of the title compound with the atom-labeling scheme (except of C and H atoms). Displacement elipsoids are drawn at the 50% probability level. Both components of the disordered ethyl group (attached to O3) are shown.

## supplementary crystallographic information

### S1. Comment

Mo_3_S_4_ clusters with diethyl dithiophosphate (dtp) ligand have been previously reported (Tang *et al.*, 2001; Yao *et al.*, 1995). In this study, complex of [Mo_3_S_4_(dtp)_4_(CH_3_CN)] (1) as starting material has been prepared by the reaction of [Mo_3_S_4_(H_2_O)_9_]^4+^ with Hdtp obtained by dissolving P_4_S_10_ in EtOH. Complex 1 reacts with acetate ion in pyridine, and then complex of [Mo_3_S_4_(dtp)_3_(µ_2_-OAc)(pyridine)] (2) was obtained. The pyridine in complex 2 loosely coordinates to the Mo_3_S_4_ core. Therefore, as estimating by VT-^1^H NMR spectroscopy and mass spectrometry, the pyridine molecule liberates from Mo_3_S_4_(dtp)_3_(µ_2_-OAc) core in solution (Hernandez-Molina *et al.*, 2011). In our material, the 4,4'-bipyridine ligand also easily liberates from Mo_3_S_4_(dtp)_3_(µ_2_-OAc) core in solution as estimated by using mass spectrometry.

In general, the metal-metal distances of the sulfur bridged complexes increase with the increase of the negative charge of the cluster center (Ogino *et al.*, 1998; Tang *et al.*, 2001). In the title compound, the Mo3—N1 distance of 2.335 (5) Å is shorter than that in2 [2.385 (9) Å], which has the same framework of [Mo_3_S_4_(dtp)_3_(µ_2_-OAc)(*L*)] with the title compound. Furthermore, the average Mo—Mo distance in the title compound [2.731[1] Å] is also shorter than that in 2 [2.738[2] Å]. This shortening of Mo—Mo distnace is probably caused by the electron donating ability of N donating atom of 4,4'-bipyridine ligand.

### S2. Experimental

The title compound was synthesized from [Mo_3_S_4_(dtp)_4_(CH_3_CN) (1) (dtp = diethyl dithiophosphate), which was prepared by the literature method (Yao *et al.*, 1995). To a solution of complex 1 (251 mg, 0.21 mmol) in 10 ml of EtOH was added excess 4,4'-bipyridine (415 mg, 2.65 mmol) and acetate anhydride (0.5 ml). After 4 h of stirring at 85°C, a brown powder was obtained (Yield: 176 mg, 70%). Red platelet single crystals of [Mo_3_S_4_(dtp)_3_(µ_2_-OAc)(4,4'-bpy)] suitable for X-ray analysis were obtained by vapor diffusion of the CHCl_3_ solution with external Et_2_O.

### S3. Refinement

All H atoms were geometrically positioned [C—H 0.95–0.99 Å] and refined as riding, with U_iso_(H) = 1.2–1.5 U_eq_ (C). The ethyl group attached to O3 was treated as disordered between two conformations with the occupancies refined to 0.65 (3) and 0.35 (3).

### Crystal data

**Table:**

[Mo_3_(C_2_H_3_O_2_)(C_4_H_10_O_2_PS_2_)_3_S_4_(C_10_H_8_N_2_)]·C_4_H_10_O	*F*(000) = 2544.00
*M**_r_* = 1261.06	*D*_x_ = 1.700 Mg m^−^^3^
Monoclinic, *P*2_1_/*c*	Mo *K*α radiation, λ = 0.71075 Å
Hall symbol: -P 2ybc	Cell parameters from 12390 reflections
*a* = 13.1573 (16) Å	θ = 3.0–27.5°
*b* = 27.647 (3) Å	µ = 1.32 mm^−^^1^
*c* = 14.2153 (17) Å	*T* = 153 K
β = 107.7183 (16)°	Platelet, red
*V* = 4925.6 (10) Å^3^	0.19 × 0.16 × 0.03 mm
*Z* = 4	

### Data collection

**Table:**

Rigaku Saturn724 diffractometer	9267 reflections with *F*^2^ > 2σ(*F*^2^)
Detector resolution: 7.111 pixels mm^-1^	*R*_int_ = 0.041
ω scans	θ_max_ = 27.5°
Absorption correction: multi-scan (*REQAB*; Rigaku, 1998)	*h* = −16→17
*T*_min_ = 0.778, *T*_max_ = 0.961	*k* = −35→35
40597 measured reflections	*l* = −18→18
11260 independent reflections	

### Refinement

**Table:**

Refinement on *F*^2^	Secondary atom site location: difference Fourier map
*R*[*F*^2^ > 2σ(*F*^2^)] = 0.040	Hydrogen site location: inferred from neighbouring sites
*wR*(*F*^2^) = 0.095	H-atom parameters constrained
*S* = 1.08	*w* = 1/[σ^2^(*F*_o_^2^) + (0.035*P*)^2^ + 9.5598*P*] where *P* = (*F*_o_^2^ + 2*F*_c_^2^)/3
11260 reflections	(Δ/σ)_max_ < 0.001
506 parameters	Δρ_max_ = 0.78 e Å^−^^3^
1 restraint	Δρ_min_ = −0.73 e Å^−^^3^
Primary atom site location: structure-invariant direct methods	

### Special details

**Table:**

Geometry. ENTER SPECIAL DETAILS OF THE MOLECULAR GEOMETRY
Refinement. Refinement was performed using all reflections. The weighted *R*-factor (*wR*) and goodness of fit (*S*) are based on *F*^2^. *R*-factor (gt) are based on *F*. The threshold expression of *F*^2^ > 2.0 σ(*F*^2^) is used only for calculating *R*-factor (gt).

### Fractional atomic coordinates and isotropic or equivalent isotropic displacement parameters (Å^2^)

**Table:**

	*x*	*y*	*z*	*U*_iso_*/*U*_eq_	Occ. (<1)
Mo2	0.10154 (2)	−0.060421 (11)	0.19523 (2)	0.01958 (8)	
Mo1	0.24940 (2)	0.009996 (11)	0.23468 (2)	0.01978 (8)	
Mo3	0.04169 (2)	0.033251 (11)	0.21877 (2)	0.01827 (7)	
S1	0.21446 (7)	−0.04305 (3)	0.10469 (6)	0.02303 (18)	
S2	0.15391 (7)	−0.01529 (3)	0.34076 (6)	0.02047 (17)	
S3	−0.03765 (7)	−0.01806 (3)	0.09060 (6)	0.02140 (18)	
S4	0.15061 (7)	0.07198 (3)	0.14388 (6)	0.02183 (18)	
S5	−0.00065 (8)	−0.10961 (4)	0.28613 (7)	0.0287 (2)	
S6	0.04814 (8)	−0.13499 (4)	0.08405 (7)	0.0290 (2)	
S7	0.35744 (8)	0.06451 (4)	0.37431 (7)	0.0276 (2)	
S8	0.40698 (8)	0.03446 (4)	0.17530 (7)	0.0302 (2)	
S9	−0.10014 (8)	0.09694 (3)	0.13671 (7)	0.02602 (19)	
S10	−0.10271 (8)	0.01686 (4)	0.30145 (7)	0.0263 (2)	
P1	−0.02992 (9)	−0.15765 (4)	0.17586 (7)	0.0280 (2)	
P2	0.46696 (8)	0.06937 (4)	0.30288 (8)	0.0290 (3)	
P3	−0.17135 (8)	0.07802 (4)	0.23600 (7)	0.0267 (2)	
O1	0.2373 (2)	−0.10679 (10)	0.28332 (19)	0.0275 (6)	
O2	0.3601 (3)	−0.04657 (10)	0.31748 (19)	0.0292 (6)	
O3	−0.0006 (3)	−0.21110 (10)	0.2143 (3)	0.0379 (7)	
O4	−0.1523 (3)	−0.16713 (11)	0.1248 (2)	0.0357 (7)	
O5	0.5800 (3)	0.05161 (11)	0.3666 (3)	0.0394 (8)	
O6	0.5017 (3)	0.12277 (11)	0.2904 (3)	0.0391 (7)	
O7	−0.1620 (3)	0.12055 (10)	0.3116 (2)	0.0349 (7)	
O8	−0.2960 (2)	0.07256 (11)	0.1910 (2)	0.0346 (7)	
O9	0.5009 (4)	0.22604 (17)	0.9179 (4)	0.0829 (14)	
N1	0.0886 (3)	0.09178 (11)	0.3430 (2)	0.0212 (6)	
N2	0.2054 (4)	0.27667 (14)	0.7073 (3)	0.0439 (10)	
C1	0.3303 (4)	−0.08972 (15)	0.3243 (3)	0.0295 (9)	
C2	0.4110 (4)	−0.12299 (17)	0.3901 (4)	0.0474 (12)	
C3	0.1102 (5)	−0.22189 (19)	0.2713 (5)	0.0655 (17)	
C4A	0.1481 (19)	−0.2603 (11)	0.239 (4)	0.122 (18)	0.35 (3)
C4B	0.1189 (9)	−0.2652 (4)	0.3227 (12)	0.081 (6)	0.65 (3)
C5	−0.2205 (4)	−0.1297 (2)	0.0645 (4)	0.0530 (14)	
C6	−0.2903 (7)	−0.1074 (4)	0.1084 (6)	0.114 (4)	
C7	0.5974 (4)	0.00443 (17)	0.4150 (4)	0.0416 (11)	
C8	0.6389 (5)	−0.0311 (2)	0.3569 (5)	0.0626 (17)	
C9	0.4255 (5)	0.15613 (19)	0.2282 (5)	0.0586 (15)	
C10	0.4810 (7)	0.1953 (3)	0.1974 (7)	0.109 (3)	
C11	−0.1951 (5)	0.1142 (2)	0.3999 (4)	0.0516 (14)	
C12	−0.1868 (6)	0.1610 (3)	0.4514 (5)	0.0699 (18)	
C13	−0.3411 (4)	0.03861 (17)	0.1090 (4)	0.0388 (11)	
C14	−0.4114 (4)	0.0658 (2)	0.0244 (4)	0.0504 (13)	
C15	0.1053 (3)	0.13808 (13)	0.3217 (3)	0.0263 (8)	
C16	0.0997 (3)	0.08171 (13)	0.4387 (3)	0.0218 (7)	
C17	0.1278 (4)	0.17474 (14)	0.3906 (3)	0.0279 (8)	
C18	0.1240 (3)	0.11629 (13)	0.5119 (3)	0.0249 (8)	
C19	0.1377 (3)	0.16418 (13)	0.4890 (3)	0.0247 (8)	
C20	0.1608 (4)	0.20292 (14)	0.5646 (3)	0.0285 (9)	
C21	0.1182 (4)	0.24874 (15)	0.5430 (3)	0.0397 (11)	
C22	0.2260 (4)	0.19456 (15)	0.6596 (3)	0.0346 (10)	
C23	0.1433 (5)	0.28370 (16)	0.6162 (4)	0.0474 (13)	
C24	0.2458 (5)	0.23228 (16)	0.7277 (3)	0.0442 (12)	
C25	0.5100 (6)	0.2665 (3)	0.9833 (6)	0.084 (2)	
C26	0.3979 (6)	0.2842 (3)	0.9759 (6)	0.091 (3)	
C27	0.6064 (8)	0.2053 (3)	0.9270 (8)	0.116 (4)	
C28	0.5875 (10)	0.1690 (4)	0.8416 (7)	0.158 (6)	
H2A	0.4733	−0.1254	0.3662	0.0569*	
H2B	0.3794	−0.1551	0.3896	0.0569*	
H2C	0.4332	−0.1102	0.4576	0.0569*	
H5A	−0.1745	−0.1045	0.0491	0.0636*	
H5B	−0.2631	−0.1444	0.0013	0.0636*	
H6A	−0.2492	−0.0916	0.1699	0.1367*	
H6B	−0.3375	−0.1318	0.1228	0.1367*	
H6C	−0.3332	−0.0832	0.0631	0.1367*	
H7A	0.5293	−0.0076	0.4223	0.0499*	
H7B	0.6491	0.0078	0.4819	0.0499*	
H8A	0.7016	−0.0175	0.3430	0.0751*	
H8B	0.5834	−0.0381	0.2947	0.0751*	
H8C	0.6590	−0.0610	0.3950	0.0751*	
H9A	0.3801	0.1389	0.1694	0.0703*	
H9B	0.3788	0.1693	0.2651	0.0703*	
H10A	0.5268	0.1822	0.1605	0.1313*	
H10B	0.5250	0.2127	0.2557	0.1313*	
H10C	0.4290	0.2176	0.1552	0.1313*	
H11A	−0.1489	0.0900	0.4441	0.0619*	
H11B	−0.2697	0.1024	0.3812	0.0619*	
H12A	−0.1120	0.1713	0.4743	0.0838*	
H12B	−0.2137	0.1576	0.5082	0.0838*	
H12C	−0.2292	0.1853	0.4060	0.0838*	
H13A	−0.3825	0.0131	0.1299	0.0465*	
H13B	−0.2830	0.0229	0.0896	0.0465*	
H14A	−0.3710	0.0922	0.0068	0.0605*	
H14B	−0.4718	0.0791	0.0426	0.0605*	
H14C	−0.4379	0.0440	−0.0322	0.0605*	
H15	0.1012	0.1458	0.2555	0.0315*	
H16	0.0903	0.0492	0.4563	0.0261*	
H17	0.1366	0.2070	0.3712	0.0334*	
H18	0.1313	0.1074	0.5782	0.0299*	
H21	0.0722	0.2561	0.4789	0.0476*	
H22	0.2570	0.1636	0.6781	0.0415*	
H23	0.1137	0.3150	0.5995	0.0569*	
H24	0.2908	0.2260	0.7928	0.0531*	
H25A	0.5511	0.2928	0.9645	0.1005*	
H25B	0.5479	0.2567	1.0520	0.1005*	
H26A	0.4027	0.3118	1.0203	0.1089*	
H26B	0.3579	0.2580	0.9949	0.1089*	
H26C	0.3612	0.2941	0.9078	0.1089*	
H27A	0.6358	0.1888	0.9913	0.1394*	
H27B	0.6569	0.2309	0.9213	0.1394*	
H28A	0.5580	0.1860	0.7786	0.1895*	
H28B	0.5371	0.1441	0.8483	0.1895*	
H28C	0.6553	0.1538	0.8433	0.1895*	
H4A	0.1555	−0.1937	0.2683	0.0786*	0.35 (3)
H4B	0.1143	−0.2270	0.3413	0.0786*	0.35 (3)
H4C	0.1542	−0.2237	0.2259	0.0786*	0.65 (3)
H4D	0.1385	−0.1953	0.3187	0.0786*	0.65 (3)
H4E	0.2220	−0.2658	0.2798	0.1462*	0.35 (3)
H4F	0.1459	−0.2553	0.1703	0.1462*	0.35 (3)
H4G	0.1047	−0.2886	0.2433	0.1462*	0.35 (3)
H4H	0.1939	−0.2712	0.3596	0.0969*	0.65 (3)
H4I	0.0922	−0.2918	0.2760	0.0969*	0.65 (3)
H4J	0.0768	−0.2634	0.3688	0.0969*	0.65 (3)

### Atomic displacement parameters (Å^2^)

**Table:**

	*U*^11^	*U*^22^	*U*^33^	*U*^12^	*U*^13^	*U*^23^
Mo2	0.01993 (15)	0.02225 (16)	0.01681 (14)	−0.00049 (12)	0.00596 (11)	−0.00109 (11)
Mo1	0.01735 (14)	0.02572 (17)	0.01616 (14)	−0.00130 (12)	0.00493 (11)	−0.00018 (11)
Mo3	0.01806 (15)	0.02209 (16)	0.01506 (13)	−0.00112 (12)	0.00562 (11)	−0.00143 (11)
S1	0.0219 (5)	0.0294 (5)	0.0194 (4)	−0.0004 (4)	0.0088 (4)	−0.0028 (4)
S2	0.0219 (5)	0.0240 (5)	0.0155 (4)	−0.0014 (4)	0.0057 (4)	−0.0005 (4)
S3	0.0203 (4)	0.0250 (5)	0.0176 (4)	−0.0009 (4)	0.0039 (4)	−0.0027 (4)
S4	0.0222 (5)	0.0261 (5)	0.0177 (4)	−0.0012 (4)	0.0068 (4)	0.0017 (4)
S5	0.0359 (6)	0.0294 (5)	0.0238 (5)	−0.0057 (5)	0.0137 (4)	−0.0007 (4)
S6	0.0360 (6)	0.0274 (5)	0.0257 (5)	−0.0042 (4)	0.0125 (4)	−0.0055 (4)
S7	0.0249 (5)	0.0362 (6)	0.0199 (4)	−0.0078 (4)	0.0040 (4)	−0.0022 (4)
S8	0.0226 (5)	0.0429 (6)	0.0274 (5)	−0.0036 (5)	0.0110 (4)	−0.0002 (4)
S9	0.0252 (5)	0.0290 (5)	0.0233 (5)	0.0040 (4)	0.0065 (4)	0.0004 (4)
S10	0.0239 (5)	0.0316 (5)	0.0270 (5)	−0.0027 (4)	0.0133 (4)	−0.0010 (4)
P1	0.0328 (6)	0.0246 (5)	0.0262 (5)	−0.0052 (5)	0.0087 (4)	−0.0005 (4)
P2	0.0211 (5)	0.0355 (6)	0.0278 (5)	−0.0059 (5)	0.0037 (4)	0.0045 (5)
P3	0.0216 (5)	0.0329 (6)	0.0265 (5)	0.0002 (4)	0.0087 (4)	−0.0072 (4)
O1	0.0259 (14)	0.0293 (14)	0.0251 (13)	0.0030 (12)	0.0042 (11)	0.0004 (11)
O2	0.0289 (15)	0.0294 (15)	0.0290 (14)	−0.0010 (12)	0.0085 (12)	0.0007 (12)
O3	0.0474 (19)	0.0248 (15)	0.0384 (17)	−0.0039 (14)	0.0086 (14)	0.0013 (13)
O4	0.0332 (16)	0.0379 (17)	0.0340 (16)	−0.0098 (13)	0.0074 (13)	0.0007 (13)
O5	0.0220 (14)	0.0494 (19)	0.0414 (17)	−0.0084 (14)	0.0016 (13)	0.0112 (15)
O6	0.0336 (16)	0.0388 (17)	0.0441 (17)	−0.0099 (14)	0.0108 (14)	0.0074 (14)
O7	0.0366 (17)	0.0388 (17)	0.0325 (15)	0.0007 (14)	0.0151 (13)	−0.0123 (13)
O8	0.0203 (14)	0.0486 (18)	0.0353 (16)	0.0021 (13)	0.0087 (12)	−0.0126 (14)
O9	0.090 (4)	0.082 (4)	0.074 (3)	−0.005 (3)	0.020 (3)	−0.024 (3)
N1	0.0240 (16)	0.0240 (16)	0.0168 (14)	−0.0003 (13)	0.0079 (12)	−0.0009 (12)
N2	0.068 (3)	0.036 (2)	0.0294 (19)	−0.015 (2)	0.0165 (19)	−0.0113 (16)
C1	0.029 (2)	0.034 (3)	0.0259 (19)	0.0043 (18)	0.0094 (16)	0.0016 (16)
C2	0.041 (3)	0.039 (3)	0.051 (3)	0.008 (3)	−0.002 (3)	0.010 (3)
C3	0.052 (4)	0.040 (3)	0.088 (5)	0.006 (3)	−0.004 (3)	0.014 (3)
C4A	0.069 (15)	0.10 (2)	0.18 (4)	0.018 (14)	0.008 (19)	−0.07 (3)
C4B	0.063 (7)	0.062 (7)	0.100 (11)	−0.003 (6)	−0.000 (7)	0.044 (7)
C5	0.039 (3)	0.066 (4)	0.051 (3)	−0.006 (3)	0.010 (3)	0.021 (3)
C6	0.099 (7)	0.156 (9)	0.093 (6)	0.073 (6)	0.039 (5)	0.056 (6)
C7	0.028 (3)	0.051 (3)	0.040 (3)	−0.005 (2)	0.0026 (19)	0.018 (2)
C8	0.049 (4)	0.070 (4)	0.080 (4)	0.022 (3)	0.037 (3)	0.034 (4)
C9	0.060 (4)	0.042 (3)	0.063 (4)	−0.002 (3)	0.004 (3)	0.019 (3)
C10	0.112 (7)	0.081 (6)	0.149 (9)	0.005 (5)	0.059 (7)	0.056 (6)
C11	0.048 (3)	0.066 (4)	0.053 (3)	−0.023 (3)	0.034 (3)	−0.033 (3)
C12	0.084 (5)	0.079 (5)	0.052 (4)	0.011 (4)	0.028 (4)	−0.030 (3)
C13	0.023 (2)	0.054 (3)	0.040 (3)	−0.003 (2)	0.0096 (18)	−0.014 (2)
C14	0.035 (3)	0.078 (4)	0.038 (3)	0.009 (3)	0.010 (2)	−0.005 (3)
C15	0.034 (2)	0.027 (2)	0.0187 (17)	−0.0028 (17)	0.0091 (15)	−0.0018 (15)
C16	0.0244 (18)	0.0238 (18)	0.0178 (16)	−0.0007 (15)	0.0076 (14)	−0.0001 (14)
C17	0.038 (3)	0.0239 (19)	0.0227 (18)	−0.0050 (17)	0.0111 (16)	−0.0010 (15)
C18	0.029 (2)	0.029 (2)	0.0179 (16)	−0.0009 (16)	0.0090 (15)	0.0010 (14)
C19	0.029 (2)	0.0255 (19)	0.0196 (17)	−0.0008 (16)	0.0076 (15)	−0.0024 (14)
C20	0.037 (3)	0.027 (2)	0.0223 (18)	−0.0045 (17)	0.0102 (17)	−0.0042 (15)
C21	0.063 (3)	0.027 (3)	0.023 (2)	0.004 (2)	0.006 (2)	−0.0006 (16)
C22	0.047 (3)	0.030 (3)	0.0241 (19)	−0.0031 (19)	0.0065 (18)	−0.0040 (16)
C23	0.078 (4)	0.026 (3)	0.036 (3)	0.003 (3)	0.015 (3)	−0.0043 (19)
C24	0.066 (4)	0.038 (3)	0.023 (2)	−0.011 (3)	0.005 (2)	−0.0072 (18)
C25	0.082 (5)	0.092 (6)	0.077 (5)	−0.017 (5)	0.024 (4)	−0.027 (4)
C26	0.082 (6)	0.105 (6)	0.085 (6)	−0.003 (5)	0.024 (5)	−0.029 (5)
C27	0.116 (8)	0.097 (7)	0.162 (10)	0.009 (6)	0.083 (7)	−0.036 (6)
C28	0.266 (15)	0.137 (9)	0.084 (7)	0.097 (10)	0.073 (8)	0.005 (6)

### Geometric parameters (Å, º)

**Table:**

Mo2—Mo1	2.6880 (5)	C20—C22	1.380 (5)
Mo2—Mo3	2.7566 (5)	C21—C23	1.384 (6)
Mo2—S1	2.2931 (11)	C22—C24	1.393 (6)
Mo2—S2	2.3332 (9)	C25—C26	1.527 (12)
Mo2—S3	2.2978 (9)	C27—C28	1.536 (14)
Mo2—S5	2.5267 (12)	C2—H2A	0.980
Mo2—S6	2.5624 (11)	C2—H2B	0.980
Mo2—O1	2.245 (3)	C2—H2C	0.980
Mo1—Mo3	2.7490 (5)	C3—H4A	0.990
Mo1—S1	2.2934 (9)	C3—H4B	0.990
Mo1—S2	2.3440 (11)	C3—H4C	0.990
Mo1—S4	2.2959 (9)	C3—H4D	0.990
Mo1—S7	2.5524 (10)	C4A—H4E	0.980
Mo1—S8	2.5561 (13)	C4A—H4F	0.980
Mo1—O2	2.216 (3)	C4A—H4G	0.980
Mo3—S2	2.3302 (9)	C4B—H4H	0.980
Mo3—S3	2.2943 (9)	C4B—H4I	0.980
Mo3—S4	2.2939 (11)	C4B—H4J	0.980
Mo3—S9	2.5726 (10)	C5—H5A	0.990
Mo3—S10	2.5604 (13)	C5—H5B	0.990
Mo3—N1	2.335 (3)	C6—H6A	0.980
S5—P1	2.0005 (15)	C6—H6B	0.980
S6—P1	1.9916 (17)	C6—H6C	0.980
S7—P2	2.0040 (18)	C7—H7A	0.990
S8—P2	1.9930 (15)	C7—H7B	0.990
S9—P3	1.9871 (17)	C8—H8A	0.980
S10—P3	2.0072 (15)	C8—H8B	0.980
P1—O3	1.582 (3)	C8—H8C	0.980
P1—O4	1.573 (3)	C9—H9A	0.990
P2—O5	1.566 (3)	C9—H9B	0.990
P2—O6	1.571 (4)	C10—H10A	0.980
P3—O7	1.572 (3)	C10—H10B	0.980
P3—O8	1.576 (3)	C10—H10C	0.980
O1—C1	1.275 (5)	C11—H11A	0.990
O2—C1	1.269 (5)	C11—H11B	0.990
O3—C3	1.467 (7)	C12—H12A	0.980
O4—C5	1.464 (6)	C12—H12B	0.980
O5—C7	1.460 (6)	C12—H12C	0.980
O6—C9	1.449 (6)	C13—H13A	0.990
O7—C11	1.459 (7)	C13—H13B	0.990
O8—C13	1.474 (6)	C14—H14A	0.980
O9—C25	1.437 (9)	C14—H14B	0.980
O9—C27	1.471 (12)	C14—H14C	0.980
N1—C15	1.348 (5)	C15—H15	0.950
N1—C16	1.353 (5)	C16—H16	0.950
N2—C23	1.319 (6)	C17—H17	0.950
N2—C24	1.334 (6)	C18—H18	0.950
C1—C2	1.498 (6)	C21—H21	0.950
C3—C4A	1.31 (4)	C22—H22	0.950
C3—C4B	1.390 (14)	C23—H23	0.950
C5—C6	1.400 (12)	C24—H24	0.950
C7—C8	1.489 (9)	C25—H25A	0.990
C9—C10	1.447 (11)	C25—H25B	0.990
C11—C12	1.476 (9)	C26—H26A	0.980
C13—C14	1.479 (7)	C26—H26B	0.980
C15—C17	1.377 (6)	C26—H26C	0.980
C16—C18	1.377 (5)	C27—H27A	0.990
C17—C19	1.397 (6)	C27—H27B	0.990
C18—C19	1.388 (6)	C28—H28A	0.980
C19—C20	1.482 (6)	C28—H28B	0.980
C20—C21	1.382 (6)	C28—H28C	0.980
			
S1···C1	3.292 (4)	C26···H2A^xi^	3.4419
S2···C1	3.163 (5)	C26···H2B^xi^	3.4125
S2···C16	3.201 (4)	C26···H9B^viii^	3.2002
S4···C15	3.317 (4)	C26···H10C^xii^	3.0682
S5···C3	3.463 (6)	C26···H17^viii^	3.3132
S6···C3	3.493 (7)	C26···H24	3.0195
S6···C5	3.464 (6)	C27···H10B^viii^	3.2606
S7···N1	3.510 (4)	C27···H14A^xvii^	3.3072
S7···C7	3.457 (5)	C27···H4H^xi^	3.4177
S7···C9	3.559 (7)	C28···H2A^ii^	3.0625
S8···C9	3.439 (6)	C28···H2B^ii^	3.4649
S9···C13	3.471 (5)	C28···H10B^viii^	3.5011
S9···C15	3.344 (4)	C28···H14A^xvii^	3.0885
S10···C11	3.423 (6)	H2A···C26^ix^	3.4419
S10···C13	3.530 (4)	H2A···C28^ii^	3.0625
S10···C16	3.309 (4)	H2A···H25A^ix^	3.3877
P1···C6	3.548 (9)	H2A···H25B^ix^	3.5015
P2···O2	3.531 (3)	H2A···H26A^ix^	2.5847
P2···C8	3.515 (6)	H2A···H28A^ii^	2.5875
P3···N1	3.315 (3)	H2A···H28B^ii^	3.0546
O1···C3	3.575 (7)	H2A···H28C^ii^	3.0464
O2···C7	3.320 (5)	H2B···C25^ix^	2.9110
O2···C8	3.563 (7)	H2B···C26^ix^	3.4125
O7···N1	3.287 (5)	H2B···C28^ii^	3.4649
O7···C15	3.508 (6)	H2B···H12B^iv^	2.9655
O7···C16	3.534 (5)	H2B···H25A^ix^	2.4602
O8···C11	3.083 (6)	H2B···H25B^ix^	2.6585
N1···C19	2.814 (5)	H2B···H26A^ix^	2.9086
N2···C20	2.809 (6)	H2B···H28A^ii^	2.8846
C15···C18	2.708 (6)	H2B···H28C^ii^	3.2018
C16···C17	2.716 (6)	H2C···S7^ii^	3.2996
C17···C21	3.011 (6)	H2C···P2^ii^	3.4451
C18···C22	3.029 (6)	H2C···O5^ii^	3.0264
C21···C24	2.690 (6)	H2C···O6^ii^	3.4373
C22···C23	2.689 (7)	H2C···C7^ii^	3.5264
S1···S3^i^	3.4702 (11)	H2C···C25^ix^	3.5350
S3···S1^i^	3.4702 (11)	H2C···H7B^ii^	3.2406
S3···S3^i^	3.1886 (14)	H2C···H11B^iv^	3.5951
S3···S4^i^	3.5392 (12)	H2C···H12B^iv^	3.3377
S4···S3^i^	3.5392 (12)	H2C···H25A^ix^	2.8835
S6···S9^i^	3.5679 (16)	H2C···H26A^ix^	2.9995
S7···C7^ii^	3.445 (5)	H5A···S4^i^	2.9932
S9···S6^i^	3.5679 (16)	H5A···H9A^i^	3.5709
O4···C21^iii^	3.457 (6)	H5A···H23^iii^	3.3284
O4···C22^iv^	3.568 (6)	H5B···S4^i^	3.5083
O4···C24^iv^	3.275 (7)	H5B···C9^i^	3.3273
O5···O8^v^	3.422 (5)	H5B···C23^iii^	3.2909
O5···C11^v^	3.335 (7)	H5B···H9A^i^	2.4505
O7···C4A^vi^	3.39 (3)	H5B···H10C^i^	3.2950
O8···O5^vii^	3.422 (5)	H5B···H23^iii^	2.9821
N2···C9^viii^	3.377 (8)	H5B···H26B^iv^	3.3870
N2···C15^viii^	3.352 (6)	H6A···H8A^vii^	3.4127
N2···C17^viii^	3.354 (7)	H6A···H8B^vii^	3.5453
C2···C25^ix^	3.537 (9)	H6A···H18^iv^	3.4690
C2···C28^ii^	3.536 (12)	H6A···H22^iv^	2.9619
C4A···O7^iii^	3.39 (3)	H6B···O9^iv^	3.3142
C7···S7^ii^	3.445 (5)	H6B···C22^iv^	3.4593
C9···N2^x^	3.377 (8)	H6B···C24^iv^	3.4818
C11···O5^vii^	3.335 (7)	H6B···H14B^xiii^	3.2176
C15···N2^x^	3.352 (6)	H6B···H22^iv^	2.8420
C17···N2^x^	3.354 (7)	H6B···H24^iv^	2.8543
C17···C26^x^	3.573 (9)	H6B···H28B^iv^	2.7998
C21···O4^vi^	3.457 (6)	H6C···S8^i^	3.4986
C22···O4^iv^	3.568 (6)	H6C···C14^xiii^	3.2438
C24···O4^iv^	3.275 (7)	H6C···H9A^i^	3.5260
C25···C2^xi^	3.537 (9)	H6C···H14B^xiii^	2.5497
C26···C17^viii^	3.573 (9)	H6C···H14C^xiii^	3.1028
C28···C2^ii^	3.536 (12)	H7A···S7^ii^	3.2294
Mo3···H15	3.2133	H7A···C7^ii^	3.2370
Mo3···H16	3.2695	H7A···H7A^ii^	2.5855
S2···H16	2.7230	H7A···H7B^ii^	3.0512
S3···H5A	2.9422	H7B···S2^ii^	3.0237
S3···H13B	3.4162	H7B···S7^ii^	2.8786
S4···H9A	3.4637	H7B···O2^ii^	3.0842
S4···H15	2.7820	H7B···C1^ii^	3.5135
S5···H5A	3.4512	H7B···H2C^ii^	3.2406
S5···H6A	3.2217	H7B···H7A^ii^	3.0512
S5···H4A	3.1626	H7B···H11B^v^	3.3117
S5···H4B	3.5647	H8A···S10^v^	2.9675
S5···H4D	2.9429	H8A···O8^v^	3.3022
S6···H5A	2.9408	H8A···C13^v^	3.5560
S6···H4A	3.0390	H8A···H6A^v^	3.4127
S6···H4C	3.2110	H8A···H11B^v^	3.3610
S6···H4D	3.5920	H8A···H13A^v^	3.0119
S7···H7A	2.9354	H8A···H16^ii^	3.4138
S7···H9B	3.3392	H8A···H18^ii^	3.2809
S8···H7A	3.5827	H8B···H6A^v^	3.5453
S8···H8B	3.1449	H8B···H13A^v^	2.8851
S8···H9A	2.9061	H8C···S7^ii^	3.3529
S9···H13B	3.0731	H8C···C16^ii^	3.3807
S9···H14A	3.4819	H8C···C18^ii^	3.1537
S9···H15	2.9956	H8C···H16^ii^	3.3483
S10···H11A	3.0522	H8C···H18^ii^	2.9601
S10···H13B	3.2214	H8C···H22^ii^	3.3243
S10···H16	2.9486	H9A···N2^x^	3.4324
P1···H5A	2.6332	H9A···C5^i^	3.3584
P1···H5B	3.3272	H9A···H5A^i^	3.5709
P1···H6A	3.3927	H9A···H5B^i^	2.4505
P1···H4A	2.5918	H9A···H6C^i^	3.5260
P1···H4B	3.1762	H9A···H14B^v^	3.4509
P1···H4C	2.9418	H9A···H23^x^	3.5746
P1···H4D	2.7158	H9B···N2^x^	2.6407
P1···H4F	3.5708	H9B···C23^x^	3.4323
P2···H7A	2.6904	H9B···C24^x^	3.1914
P2···H7B	3.3771	H9B···C25^x^	3.5346
P2···H8B	3.3602	H9B···C26^x^	3.2002
P2···H9A	2.6980	H9B···H23^x^	3.5917
P2···H9B	2.9811	H9B···H24^x^	3.1866
P3···H11A	2.9004	H9B···H25A^x^	3.2194
P3···H11B	2.8305	H9B···H26A^x^	3.5830
P3···H13A	3.2653	H9B···H26C^x^	2.3416
P3···H13B	2.6366	H10A···O9^xiv^	3.5739
P3···H14A	3.5300	H10A···C25^xiv^	3.3903
O1···H2A	3.0104	H10A···H14B^v^	3.3083
O1···H2B	2.4175	H10A···H25B^xiv^	2.6381
O1···H2C	2.9837	H10A···H26B^xiv^	3.4193
O1···H4A	2.6147	H10A···H27A^xiv^	3.1581
O1···H4C	3.4296	H10A···H4E^xviii^	3.4652
O1···H4D	2.8860	H10B···O9^x^	2.9543
O2···H2A	2.6109	H10B···C25^x^	3.3501
O2···H2B	3.1567	H10B···C27^x^	3.2606
O2···H2C	2.6124	H10B···C28^x^	3.5011
O2···H7A	2.5136	H10B···H12C^v^	3.3740
O2···H8B	3.0616	H10B···H25A^x^	2.8861
O3···H4E	3.1739	H10B···H25B^xiv^	3.2389
O3···H4F	2.5160	H10B···H26C^x^	3.4916
O3···H4G	2.5157	H10B···H27B^x^	2.9158
O3···H4H	3.2180	H10B···H28A^x^	2.8396
O3···H4I	2.5652	H10B···H4E^xviii^	3.5649
O3···H4J	2.5671	H10C···N2^x^	3.2493
O4···H6A	2.6253	H10C···C24^x^	3.2106
O4···H6B	2.6180	H10C···C25^xiv^	3.2431
O4···H6C	3.2490	H10C···C26^xiv^	3.0682
O5···H8A	2.5785	H10C···H5B^i^	3.2950
O5···H8B	2.6873	H10C···H24^x^	3.4249
O5···H8C	3.2678	H10C···H25B^xiv^	2.6762
O6···H10A	2.5660	H10C···H26A^xiv^	3.1897
O6···H10B	2.5700	H10C···H26B^xiv^	2.4528
O6···H10C	3.2198	H10C···H28A^x^	3.3548
O7···H12A	2.6117	H11A···O5^vii^	3.5596
O7···H12B	3.2380	H11B···P2^vii^	3.4255
O7···H12C	2.5526	H11B···O5^vii^	2.3830
O8···H11A	3.5609	H11B···O6^vii^	2.9446
O8···H11B	2.7456	H11B···C7^vii^	3.3374
O8···H14A	2.5601	H11B···H2C^iv^	3.5951
O8···H14B	2.6201	H11B···H7B^vii^	3.3117
O8···H14C	3.2509	H11B···H8A^vii^	3.3610
O9···H26A	3.2488	H12A···C4A^vi^	3.4824
O9···H26B	2.6025	H12A···H4B^iv^	3.0483
O9···H26C	2.6039	H12A···H4D^iv^	3.1385
O9···H28A	2.5719	H12A···H4F^vi^	2.8264
O9···H28B	2.5736	H12A···H4G^vi^	3.3149
O9···H28C	3.2484	H12A···H4J^iv^	3.3231
N1···H17	3.2480	H12B···O1^iv^	3.3780
N1···H18	3.2467	H12B···C2^iv^	3.4677
N2···H21	3.2359	H12B···C3^iv^	3.4984
N2···H22	3.2521	H12B···H2B^iv^	2.9655
C1···H7A	3.4251	H12B···H2C^iv^	3.3377
C2···H7A	3.5176	H12B···H25A^xv^	3.2661
C6···H13A	3.5913	H12B···H27B^xv^	3.5573
C8···H2A	3.4267	H12B···H4A^iv^	3.1926
C13···H6C	3.4373	H12B···H4B^iv^	2.8741
C15···H16	3.1563	H12B···H4D^iv^	2.5766
C16···H11A	3.3031	H12B···H4J^iv^	3.5966
C16···H15	3.1554	H12C···C4A^vi^	2.9987
C17···H18	3.2425	H12C···H10B^vii^	3.3740
C17···H21	2.7810	H12C···H25A^xv^	3.2975
C18···H11A	3.4988	H12C···H27B^xv^	2.8033
C18···H12A	3.3503	H12C···H4C^vi^	3.4564
C18···H17	3.2431	H12C···H4E^vi^	2.9933
C18···H22	2.7994	H12C···H4F^vi^	2.4081
C19···H12A	3.2332	H12C···H4G^vi^	3.1346
C19···H15	3.2458	H13A···S8^vii^	3.0855
C19···H16	3.2446	H13A···C8^vii^	3.3811
C19···H21	2.6737	H13A···H8A^vii^	3.0119
C19···H22	2.6685	H13A···H8B^vii^	2.8851
C20···H12A	3.5347	H13A···H14C^xiii^	2.8307
C20···H17	2.6721	H13B···S1^i^	3.2034
C20···H18	2.6857	H14A···S1^i^	3.2546
C20···H23	3.2290	H14A···C27^xvi^	3.3072
C20···H24	3.2359	H14A···C28^xvi^	3.0885
C21···H12A	3.5930	H14A···H27A^xvi^	2.6815
C21···H17	2.7778	H14A···H28B^xvi^	2.6335
C21···H22	3.2294	H14A···H28C^xvi^	2.9842
C22···H18	2.7973	H14B···S8^vii^	3.0751
C22···H21	3.2287	H14B···C6^xiii^	3.3045
C23···H24	3.1060	H14B···H6B^xiii^	3.2176
C24···H23	3.1063	H14B···H6C^xiii^	2.5497
C25···H27A	2.6934	H14B···H9A^vii^	3.4509
C25···H27B	2.5540	H14B···H10A^vii^	3.3083
C27···H25A	2.6281	H14B···H14C^xiii^	3.5946
C27···H25B	2.5694	H14B···H27A^xvi^	3.5141
H2A···H7A	3.3802	H14B···H28B^xvi^	3.3266
H2A···H8B	3.1406	H14C···S1^i^	3.3983
H2A···H8C	2.9492	H14C···S8^i^	3.0848
H2B···H4A	3.1145	H14C···C13^xiii^	3.5963
H2B···H4D	3.2178	H14C···H6C^xiii^	3.1028
H2C···H7A	3.2072	H14C···H13A^xiii^	2.8307
H5A···H6A	2.2512	H14C···H14B^xiii^	3.5946
H5A···H6B	2.7636	H14C···H14C^xiii^	3.2167
H5A···H6C	2.2374	H14C···H28B^xvi^	3.2106
H5B···H6A	2.7634	H15···N2^x^	2.7404
H5B···H6B	2.2571	H15···C23^x^	2.9484
H5B···H6C	2.2315	H15···H23^x^	2.5171
H6A···H13A	3.3442	H15···H4G^vi^	3.2644
H6A···H13B	3.3495	H15···H4I^vi^	2.9924
H6C···H13A	2.9644	H15···H4J^vi^	3.5197
H6C···H13B	3.0048	H16···H8A^ii^	3.4138
H7A···H8A	2.8354	H16···H8C^ii^	3.3483
H7A···H8B	2.3013	H17···O3^vi^	2.9130
H7A···H8C	2.3752	H17···O4^vi^	3.4859
H7B···H8A	2.3859	H17···N2^x^	2.7812
H7B···H8B	2.8342	H17···C24^x^	3.2904
H7B···H8C	2.2918	H17···C26^x^	3.3132
H9A···H10A	2.3072	H17···H24^x^	3.1885
H9A···H10B	2.8077	H17···H26A^x^	3.5352
H9A···H10C	2.2966	H17···H26B^x^	3.0637
H9B···H10A	2.8076	H17···H26C^x^	2.8398
H9B···H10B	2.3043	H17···H4G^vi^	3.0989
H9B···H10C	2.2995	H17···H4I^vi^	3.0996
H11A···H12A	2.3120	H18···S5^iv^	2.9490
H11A···H12B	2.3511	H18···C8^ii^	3.5701
H11A···H12C	2.8306	H18···H6A^iv^	3.4690
H11A···H16	3.2978	H18···H8A^ii^	3.2809
H11B···H12A	2.8302	H18···H8C^ii^	2.9601
H11B···H12B	2.3071	H21···S6^vi^	3.3923
H11B···H12C	2.3563	H21···P1^vi^	3.1759
H12A···H18	3.5605	H21···O3^vi^	2.7702
H12A···H21	3.3595	H21···O4^vi^	2.9537
H13A···H14A	2.8340	H21···C4A^vi^	3.5705
H13A···H14B	2.3162	H21···H4C^vi^	3.5188
H13A···H14C	2.3559	H21···H4F^vi^	3.0214
H13B···H14A	2.3604	H21···H4G^vi^	3.5283
H13B···H14B	2.8337	H21···H4J^iv^	3.3398
H13B···H14C	2.3117	H22···O4^iv^	3.4800
H15···H17	2.3051	H22···C6^iv^	3.3176
H16···H18	2.3041	H22···H6A^iv^	2.9619
H17···H21	2.3865	H22···H6B^iv^	2.8420
H18···H22	2.3952	H22···H8C^ii^	3.3243
H21···H23	2.3067	H22···H26A^x^	3.4343
H22···H24	2.3215	H23···S4^viii^	3.1943
H25A···H26A	2.3787	H23···S6^vi^	3.1414
H25A···H26B	2.8712	H23···O4^vi^	3.4132
H25A···H26C	2.3814	H23···C5^vi^	3.4274
H25A···H27A	3.0660	H23···C15^viii^	3.4464
H25A···H27B	2.4023	H23···H5A^vi^	3.3284
H25B···H26A	2.3777	H23···H5B^vi^	2.9821
H25B···H26B	2.3825	H23···H9A^viii^	3.5746
H25B···H26C	2.8712	H23···H9B^viii^	3.5917
H25B···H27A	2.4917	H23···H15^viii^	2.5171
H25B···H27B	2.7642	H23···H4J^iv^	3.0345
H27A···H28A	2.8837	H24···O4^iv^	2.9402
H27A···H28B	2.3953	H24···O9	2.7956
H27A···H28C	2.4001	H24···C6^iv^	3.5677
H27B···H28A	2.3962	H24···C25	3.4865
H27B···H28B	2.8838	H24···C26	3.0195
H27B···H28C	2.3991	H24···H6B^iv^	2.8543
H4A···H4E	2.1634	H24···H9B^viii^	3.1866
H4A···H4F	2.1791	H24···H10C^viii^	3.4249
H4A···H4G	2.7034	H24···H17^viii^	3.1885
H4B···H4E	2.1634	H24···H26B	2.8758
H4B···H4F	2.7036	H24···H26C	2.4808
H4B···H4G	2.1794	H25A···O6^viii^	3.3194
H4C···H4H	2.2368	H25A···C2^xi^	3.0510
H4C···H4I	2.2514	H25A···C9^viii^	3.5543
H4C···H4J	2.7617	H25A···H2A^xi^	3.3877
H4D···H4H	2.2384	H25A···H2B^xi^	2.4602
H4D···H4I	2.7619	H25A···H2C^xi^	2.8835
H4D···H4J	2.2499	H25A···H9B^viii^	3.2194
S1···H13B^i^	3.2034	H25A···H10B^viii^	2.8861
S1···H14A^i^	3.2546	H25A···H12B^xix^	3.2661
S1···H14C^i^	3.3983	H25A···H12C^xix^	3.2975
S1···H28C^ii^	3.4759	H25B···C2^xi^	3.4306
S2···H7B^ii^	3.0237	H25B···C10^xii^	3.0055
S4···H5A^i^	2.9932	H25B···H2A^xi^	3.5015
S4···H5B^i^	3.5083	H25B···H2B^xi^	2.6585
S4···H23^x^	3.1943	H25B···H10A^xii^	2.6381
S5···H18^iv^	2.9490	H25B···H10B^xii^	3.2389
S6···H21^iii^	3.3923	H25B···H10C^xii^	2.6762
S6···H23^iii^	3.1414	H25B···H28A^viii^	3.5568
S7···H2C^ii^	3.2996	H25B···H4E^xi^	3.2955
S7···H7A^ii^	3.2294	H25B···H4H^xi^	3.3334
S7···H7B^ii^	2.8786	H26A···C2^xi^	2.9966
S7···H8C^ii^	3.3529	H26A···C17^viii^	3.5438
S8···H6C^i^	3.4986	H26A···C19^viii^	3.4432
S8···H13A^v^	3.0855	H26A···C20^viii^	3.4517
S8···H14B^v^	3.0751	H26A···C22^viii^	3.4868
S8···H14C^i^	3.0848	H26A···H2A^xi^	2.5847
S9···H4I^vi^	3.3077	H26A···H2B^xi^	2.9086
S10···H8A^vii^	2.9675	H26A···H2C^xi^	2.9995
P1···H21^iii^	3.1759	H26A···H9B^viii^	3.5830
P2···H2C^ii^	3.4451	H26A···H10C^xii^	3.1897
P2···H11B^v^	3.4255	H26A···H17^viii^	3.5352
O1···H12B^iv^	3.3780	H26A···H22^viii^	3.4343
O1···H28C^ii^	2.9104	H26B···C10^xii^	3.3290
O2···H7B^ii^	3.0842	H26B···C17^viii^	3.4767
O3···H17^iii^	2.9130	H26B···C19^viii^	3.5897
O3···H21^iii^	2.7702	H26B···C20^viii^	3.2312
O4···H17^iii^	3.4859	H26B···C21^viii^	3.4303
O4···H21^iii^	2.9537	H26B···C22^viii^	3.5617
O4···H22^iv^	3.4800	H26B···H5B^iv^	3.3870
O4···H23^iii^	3.4132	H26B···H10A^xii^	3.4193
O4···H24^iv^	2.9402	H26B···H10C^xii^	2.4528
O5···H2C^ii^	3.0264	H26B···H17^viii^	3.0637
O5···H11A^v^	3.5596	H26B···H24	2.8758
O5···H11B^v^	2.3830	H26C···N2	3.0003
O6···H2C^ii^	3.4373	H26C···C9^viii^	3.2319
O6···H11B^v^	2.9446	H26C···C17^viii^	3.1259
O6···H25A^x^	3.3194	H26C···C24	3.0682
O7···H4E^vi^	3.4004	H26C···H9B^viii^	2.3416
O7···H4F^vi^	3.4448	H26C···H10B^viii^	3.4916
O7···H4G^vi^	2.8015	H26C···H17^viii^	2.8398
O7···H4I^vi^	2.9944	H26C···H24	2.4808
O8···H8A^vii^	3.3022	H27A···C14^xvii^	3.5130
O9···H6B^iv^	3.3142	H27A···H10A^xii^	3.1581
O9···H10A^xii^	3.5739	H27A···H14A^xvii^	2.6815
O9···H10B^viii^	2.9543	H27A···H14B^xvii^	3.5141
O9···H24	2.7956	H27A···H4E^xi^	3.4585
N2···H9A^viii^	3.4324	H27A···H4H^xi^	2.8018
N2···H9B^viii^	2.6407	H27B···C12^xix^	3.5788
N2···H10C^viii^	3.2493	H27B···H10B^viii^	2.9158
N2···H15^viii^	2.7404	H27B···H12B^xix^	3.5573
N2···H17^viii^	2.7812	H27B···H12C^xix^	2.8033
N2···H26C	3.0003	H27B···H4F^ii^	3.3072
N2···H4J^iv^	3.5556	H27B···H4H^xi^	3.1365
C1···H7B^ii^	3.5135	H28A···C1^ii^	3.5623
C1···H28A^ii^	3.5623	H28A···C2^ii^	3.0896
C1···H28C^ii^	3.0206	H28A···C10^viii^	3.5244
C2···H12B^iv^	3.4677	H28A···H2A^ii^	2.5875
C2···H25A^ix^	3.0510	H28A···H2B^ii^	2.8846
C2···H25B^ix^	3.4306	H28A···H10B^viii^	2.8396
C2···H26A^ix^	2.9966	H28A···H10C^viii^	3.3548
C2···H28A^ii^	3.0896	H28A···H25B^x^	3.5568
C2···H28C^ii^	3.2764	H28B···C14^xvii^	3.2218
C3···H12B^iv^	3.4984	H28B···H2A^ii^	3.0546
C4A···H12A^iii^	3.4824	H28B···H6B^iv^	2.7998
C4A···H12C^iii^	2.9987	H28B···H14A^xvii^	2.6335
C4A···H21^iii^	3.5705	H28B···H14B^xvii^	3.3266
C5···H9A^i^	3.3584	H28B···H14C^xvii^	3.2106
C5···H23^iii^	3.4274	H28C···S1^ii^	3.4759
C6···H14B^xiii^	3.3045	H28C···O1^ii^	2.9104
C6···H22^iv^	3.3176	H28C···C1^ii^	3.0206
C6···H24^iv^	3.5677	H28C···C2^ii^	3.2764
C7···H2C^ii^	3.5264	H28C···H2A^ii^	3.0464
C7···H7A^ii^	3.2370	H28C···H2B^ii^	3.2018
C7···H11B^v^	3.3374	H28C···H14A^xvii^	2.9842
C8···H13A^v^	3.3811	H28C···H4A^ii^	3.5126
C8···H18^ii^	3.5701	H28C···H4C^ii^	3.5341
C9···H5B^i^	3.3273	H4A···H12B^iv^	3.1926
C9···H25A^x^	3.5543	H4A···H28C^ii^	3.5126
C9···H26C^x^	3.2319	H4B···C12^iv^	3.3469
C10···H25B^xiv^	3.0055	H4B···H12A^iv^	3.0483
C10···H26B^xiv^	3.3290	H4B···H12B^iv^	2.8741
C10···H28A^x^	3.5244	H4C···H12C^iii^	3.4564
C12···H27B^xv^	3.5788	H4C···H21^iii^	3.5188
C12···H4B^iv^	3.3469	H4C···H28C^ii^	3.5341
C12···H4D^iv^	3.2737	H4D···C12^iv^	3.2737
C12···H4F^vi^	3.0341	H4D···H12A^iv^	3.1385
C12···H4G^vi^	3.5485	H4D···H12B^iv^	2.5766
C13···H8A^vii^	3.5560	H4E···O7^iii^	3.4004
C13···H14C^xiii^	3.5963	H4E···H10A^xx^	3.4652
C14···H6C^xiii^	3.2438	H4E···H10B^xx^	3.5649
C14···H27A^xvi^	3.5130	H4E···H12C^iii^	2.9933
C14···H28B^xvi^	3.2218	H4E···H25B^ix^	3.2955
C15···H23^x^	3.4464	H4E···H27A^ix^	3.4585
C15···H4G^vi^	3.3235	H4F···O7^iii^	3.4448
C15···H4I^vi^	3.2002	H4F···C12^iii^	3.0341
C16···H8C^ii^	3.3807	H4F···H12A^iii^	2.8264
C17···H26A^x^	3.5438	H4F···H12C^iii^	2.4081
C17···H26B^x^	3.4767	H4F···H21^iii^	3.0214
C17···H26C^x^	3.1259	H4F···H27B^ii^	3.3072
C17···H4G^vi^	3.2354	H4G···O7^iii^	2.8015
C17···H4I^vi^	3.2663	H4G···C12^iii^	3.5485
C18···H8C^ii^	3.1537	H4G···C15^iii^	3.3235
C19···H26A^x^	3.4432	H4G···C17^iii^	3.2354
C19···H26B^x^	3.5897	H4G···H12A^iii^	3.3149
C20···H26A^x^	3.4517	H4G···H12C^iii^	3.1346
C20···H26B^x^	3.2312	H4G···H15^iii^	3.2644
C21···H26B^x^	3.4303	H4G···H17^iii^	3.0989
C21···H4J^iv^	3.2046	H4G···H21^iii^	3.5283
C22···H6B^iv^	3.4593	H4H···C27^ix^	3.4177
C22···H26A^x^	3.4868	H4H···H25B^ix^	3.3334
C22···H26B^x^	3.5617	H4H···H27A^ix^	2.8018
C23···H5B^vi^	3.2909	H4H···H27B^ix^	3.1365
C23···H9B^viii^	3.4323	H4I···S9^iii^	3.3077
C23···H15^viii^	2.9484	H4I···O7^iii^	2.9944
C23···H4J^iv^	3.0201	H4I···C15^iii^	3.2002
C24···H6B^iv^	3.4818	H4I···C17^iii^	3.2663
C24···H9B^viii^	3.1914	H4I···H15^iii^	2.9924
C24···H10C^viii^	3.2106	H4I···H17^iii^	3.0996
C24···H17^viii^	3.2904	H4J···N2^iv^	3.5556
C24···H26C	3.0682	H4J···C21^iv^	3.2046
C25···H2B^xi^	2.9110	H4J···C23^iv^	3.0201
C25···H2C^xi^	3.5350	H4J···H12A^iv^	3.3231
C25···H9B^viii^	3.5346	H4J···H12B^iv^	3.5966
C25···H10A^xii^	3.3903	H4J···H15^iii^	3.5197
C25···H10B^viii^	3.3501	H4J···H21^iv^	3.3398
C25···H10C^xii^	3.2431	H4J···H23^iv^	3.0345
C25···H24	3.4865		
			
Mo1—Mo2—Mo3	60.634 (13)	N1—C15—C17	123.6 (4)
Mo1—Mo2—S1	54.13 (3)	N1—C16—C18	123.1 (4)
Mo1—Mo2—S2	55.11 (3)	C15—C17—C19	119.5 (4)
Mo1—Mo2—S3	98.60 (3)	C16—C18—C19	120.1 (4)
Mo1—Mo2—S5	138.34 (3)	C17—C19—C18	117.1 (4)
Mo1—Mo2—S6	139.16 (3)	C17—C19—C20	120.8 (4)
Mo1—Mo2—O1	83.95 (7)	C18—C19—C20	122.1 (4)
Mo3—Mo2—S1	97.42 (3)	C19—C20—C21	121.4 (4)
Mo3—Mo2—S2	53.71 (3)	C19—C20—C22	121.2 (4)
Mo3—Mo2—S3	53.05 (3)	C21—C20—C22	117.4 (4)
Mo3—Mo2—S5	103.14 (3)	C20—C21—C23	119.0 (4)
Mo3—Mo2—S6	142.74 (3)	C20—C22—C24	118.9 (4)
Mo3—Mo2—O1	132.60 (7)	N2—C23—C21	124.8 (5)
S1—Mo2—S2	108.58 (4)	N2—C24—C22	124.1 (4)
S1—Mo2—S3	93.14 (4)	O9—C25—C26	108.4 (6)
S1—Mo2—S5	159.43 (4)	O9—C27—C28	105.3 (8)
S1—Mo2—S6	85.71 (4)	C1—C2—H2A	109.469
S1—Mo2—O1	83.75 (9)	C1—C2—H2B	109.470
S2—Mo2—S3	105.28 (4)	C1—C2—H2C	109.476
S2—Mo2—S5	83.91 (4)	H2A—C2—H2B	109.470
S2—Mo2—S6	158.23 (4)	H2A—C2—H2C	109.479
S2—Mo2—O1	80.83 (8)	H2B—C2—H2C	109.462
S3—Mo2—S5	99.32 (4)	O3—C3—H4A	108.998
S3—Mo2—S6	89.77 (3)	O3—C3—H4B	108.979
S3—Mo2—O1	173.80 (8)	O3—C3—H4C	109.217
S5—Mo2—S6	78.09 (4)	O3—C3—H4D	109.218
S5—Mo2—O1	82.21 (9)	C4A—C3—H4A	108.975
S6—Mo2—O1	84.66 (7)	C4A—C3—H4B	108.979
Mo2—Mo1—Mo3	60.919 (13)	C4B—C3—H4C	109.203
Mo2—Mo1—S1	54.12 (3)	C4B—C3—H4D	109.211
Mo2—Mo1—S2	54.73 (3)	H4A—C3—H4B	107.773
Mo2—Mo1—S4	99.98 (3)	H4C—C3—H4D	107.903
Mo2—Mo1—S7	141.85 (3)	C3—C4A—H4E	109.471
Mo2—Mo1—S8	136.97 (3)	C3—C4A—H4F	109.474
Mo2—Mo1—O2	85.23 (8)	C3—C4A—H4G	109.473
Mo3—Mo1—S1	97.63 (3)	H4E—C4A—H4F	109.471
Mo3—Mo1—S2	53.74 (3)	H4E—C4A—H4G	109.468
Mo3—Mo1—S4	53.17 (3)	H4F—C4A—H4G	109.470
Mo3—Mo1—S7	103.32 (3)	C3—C4B—H4H	109.468
Mo3—Mo1—S8	142.84 (3)	C3—C4B—H4I	109.477
Mo3—Mo1—O2	133.25 (9)	C3—C4B—H4J	109.470
S1—Mo1—S2	108.20 (4)	H4H—C4B—H4I	109.470
S1—Mo1—S4	94.82 (3)	H4H—C4B—H4J	109.468
S1—Mo1—S7	158.72 (4)	H4I—C4B—H4J	109.473
S1—Mo1—S8	83.51 (4)	O4—C5—H5A	108.557
S1—Mo1—O2	85.12 (8)	O4—C5—H5B	108.563
S2—Mo1—S4	105.17 (4)	C6—C5—H5A	108.557
S2—Mo1—S7	87.51 (4)	C6—C5—H5B	108.561
S2—Mo1—S8	159.85 (3)	H5A—C5—H5B	107.550
S2—Mo1—O2	80.93 (9)	C5—C6—H6A	109.482
S4—Mo1—S7	94.73 (4)	C5—C6—H6B	109.466
S4—Mo1—S8	89.67 (4)	C5—C6—H6C	109.472
S4—Mo1—O2	173.54 (9)	H6A—C6—H6B	109.464
S7—Mo1—S8	77.57 (4)	H6A—C6—H6C	109.469
S7—Mo1—O2	83.29 (7)	H6B—C6—H6C	109.474
S8—Mo1—O2	83.91 (9)	O5—C7—H7A	109.431
Mo2—Mo3—Mo1	58.447 (11)	O5—C7—H7B	109.426
Mo2—Mo3—S2	53.81 (3)	C8—C7—H7A	109.435
Mo2—Mo3—S3	53.17 (3)	C8—C7—H7B	109.437
Mo2—Mo3—S4	98.06 (3)	H7A—C7—H7B	108.034
Mo2—Mo3—S9	141.49 (3)	C7—C8—H8A	109.485
Mo2—Mo3—S10	99.71 (3)	C7—C8—H8B	109.480
Mo2—Mo3—N1	136.32 (7)	C7—C8—H8C	109.469
Mo1—Mo3—S2	54.21 (3)	H8A—C8—H8B	109.472
Mo1—Mo3—S3	96.97 (3)	H8A—C8—H8C	109.453
Mo1—Mo3—S4	53.24 (3)	H8B—C8—H8C	109.469
Mo1—Mo3—S9	139.74 (3)	O6—C9—H9A	109.657
Mo1—Mo3—S10	141.15 (3)	O6—C9—H9B	109.669
Mo1—Mo3—N1	94.15 (8)	C10—C9—H9A	109.670
S2—Mo3—S3	105.49 (4)	C10—C9—H9B	109.662
S2—Mo3—S4	105.68 (4)	H9A—C9—H9B	108.176
S2—Mo3—S9	159.97 (4)	C9—C10—H10A	109.481
S2—Mo3—S10	86.97 (4)	C9—C10—H10B	109.483
S2—Mo3—N1	82.79 (8)	C9—C10—H10C	109.474
S3—Mo3—S4	96.75 (4)	H10A—C10—H10B	109.465
S3—Mo3—S9	88.35 (3)	H10A—C10—H10C	109.461
S3—Mo3—S10	91.91 (4)	H10B—C10—H10C	109.464
S3—Mo3—N1	168.68 (8)	O7—C11—H11A	109.893
S4—Mo3—S9	86.53 (4)	O7—C11—H11B	109.888
S4—Mo3—S10	162.05 (4)	C12—C11—H11A	109.884
S4—Mo3—N1	88.20 (9)	C12—C11—H11B	109.887
S9—Mo3—S10	78.00 (4)	H11A—C11—H11B	108.302
S9—Mo3—N1	81.78 (8)	C11—C12—H12A	109.462
S10—Mo3—N1	80.68 (9)	C11—C12—H12B	109.471
Mo2—S1—Mo1	71.76 (3)	C11—C12—H12C	109.464
Mo2—S2—Mo1	70.16 (3)	H12A—C12—H12B	109.476
Mo2—S2—Mo3	72.47 (3)	H12A—C12—H12C	109.477
Mo1—S2—Mo3	72.05 (3)	H12B—C12—H12C	109.477
Mo2—S3—Mo3	73.78 (3)	O8—C13—H13A	109.952
Mo1—S4—Mo3	73.59 (3)	O8—C13—H13B	109.941
Mo2—S5—P1	87.83 (6)	C14—C13—H13A	109.942
Mo2—S6—P1	87.03 (5)	C14—C13—H13B	109.938
Mo1—S7—P2	87.81 (5)	H13A—C13—H13B	108.342
Mo1—S8—P2	87.94 (6)	C13—C14—H14A	109.474
Mo3—S9—P3	86.07 (5)	C13—C14—H14B	109.477
Mo3—S10—P3	86.00 (6)	C13—C14—H14C	109.471
S5—P1—S6	106.85 (7)	H14A—C14—H14B	109.467
S5—P1—O3	112.41 (13)	H14A—C14—H14C	109.465
S5—P1—O4	113.48 (14)	H14B—C14—H14C	109.474
S6—P1—O3	113.57 (16)	N1—C15—H15	118.190
S6—P1—O4	114.14 (13)	C17—C15—H15	118.198
O3—P1—O4	96.40 (17)	N1—C16—H16	118.428
S7—P2—S8	106.38 (7)	C18—C16—H16	118.430
S7—P2—O5	112.85 (15)	C15—C17—H17	120.245
S7—P2—O6	113.51 (15)	C19—C17—H17	120.233
S8—P2—O5	114.71 (14)	C16—C18—H18	119.959
S8—P2—O6	113.61 (14)	C19—C18—H18	119.951
O5—P2—O6	95.87 (16)	C20—C21—H21	120.488
S9—P3—S10	107.94 (7)	C23—C21—H21	120.495
S9—P3—O7	109.43 (14)	C20—C22—H22	120.555
S9—P3—O8	113.24 (13)	C24—C22—H22	120.546
S10—P3—O7	112.49 (12)	N2—C23—H23	117.622
S10—P3—O8	111.92 (13)	C21—C23—H23	117.625
O7—P3—O8	101.80 (17)	N2—C24—H24	117.965
Mo2—O1—C1	122.2 (3)	C22—C24—H24	117.978
Mo1—O2—C1	122.4 (3)	O9—C25—H25A	110.009
P1—O3—C3	118.5 (3)	O9—C25—H25B	110.010
P1—O4—C5	120.8 (3)	C26—C25—H25A	110.003
P2—O5—C7	122.5 (3)	C26—C25—H25B	110.012
P2—O6—C9	119.7 (3)	H25A—C25—H25B	108.368
P3—O7—C11	121.1 (3)	C25—C26—H26A	109.471
P3—O8—C13	120.0 (3)	C25—C26—H26B	109.467
C25—O9—C27	110.9 (6)	C25—C26—H26C	109.468
Mo3—N1—C15	120.8 (3)	H26A—C26—H26B	109.475
Mo3—N1—C16	122.7 (3)	H26A—C26—H26C	109.474
C15—N1—C16	116.5 (3)	H26B—C26—H26C	109.472
C23—N2—C24	115.8 (4)	O9—C27—H27A	110.693
O1—C1—O2	126.1 (4)	O9—C27—H27B	110.669
O1—C1—C2	117.2 (4)	C28—C27—H27A	110.702
O2—C1—C2	116.6 (4)	C28—C27—H27B	110.678
O3—C3—C4A	113.0 (13)	H27A—C27—H27B	108.793
O3—C3—C4B	112.0 (7)	C27—C28—H28A	109.477
O4—C5—C6	114.8 (6)	C27—C28—H28B	109.468
O5—C7—C8	111.0 (5)	C27—C28—H28C	109.474
O6—C9—C10	110.0 (5)	H28A—C28—H28B	109.480
O7—C11—C12	109.0 (5)	H28A—C28—H28C	109.477
O8—C13—C14	108.7 (4)	H28B—C28—H28C	109.451
			
Mo1—Mo2—Mo3—Mo1	0.0	S4—Mo1—Mo3—S10	−164.92 (4)
Mo1—Mo2—Mo3—S2	−66.4	S4—Mo1—Mo3—N1	−84.64 (3)
Mo1—Mo2—Mo3—S3	129.640 (14)	Mo3—Mo1—S7—P2	−145.52 (3)
Mo1—Mo2—Mo3—S4	37.218 (11)	S7—Mo1—Mo3—Mo2	−142.47 (3)
Mo1—Mo2—Mo3—S9	131.80 (3)	S7—Mo1—Mo3—S2	−76.76 (3)
Mo1—Mo2—Mo3—S10	−145.299 (13)	S7—Mo1—Mo3—S3	179.15 (3)
Mo1—Mo2—Mo3—N1	−58.68 (14)	S7—Mo1—Mo3—S4	85.91 (3)
Mo3—Mo2—Mo1—Mo3	0.0	S7—Mo1—Mo3—S9	83.44 (4)
Mo3—Mo2—Mo1—S1	−126.147 (12)	S7—Mo1—Mo3—S10	−79.01 (4)
Mo3—Mo2—Mo1—S2	64.2	S7—Mo1—Mo3—N1	1.27 (3)
Mo3—Mo2—Mo1—S4	−37.414 (12)	Mo3—Mo1—S8—P2	99.41 (4)
Mo3—Mo2—Mo1—S7	73.66 (2)	S8—Mo1—Mo3—Mo2	130.62 (5)
Mo3—Mo2—Mo1—S8	−137.777 (19)	S8—Mo1—Mo3—S2	−163.68 (5)
Mo3—Mo2—Mo1—O2	146.435 (14)	S8—Mo1—Mo3—S3	92.23 (5)
Mo1—Mo2—S1—Mo1	0.0	S8—Mo1—Mo3—S4	−1.01 (5)
S1—Mo2—Mo1—Mo3	126.15 (3)	S8—Mo1—Mo3—S9	−3.48 (5)
S1—Mo2—Mo1—S1	0.00 (3)	S8—Mo1—Mo3—S10	−165.92 (4)
S1—Mo2—Mo1—S2	−169.67 (3)	S8—Mo1—Mo3—N1	−85.64 (5)
S1—Mo2—Mo1—S4	88.73 (3)	Mo3—Mo1—O2—C1	41.1 (3)
S1—Mo2—Mo1—S7	−160.19 (4)	O2—Mo1—Mo3—Mo2	−49.16 (10)
S1—Mo2—Mo1—S8	−11.63 (3)	O2—Mo1—Mo3—S2	16.55 (10)
S1—Mo2—Mo1—O2	−87.42 (3)	O2—Mo1—Mo3—S3	−87.54 (10)
Mo1—Mo2—S2—Mo1	−0.0	O2—Mo1—Mo3—S4	179.22 (10)
Mo1—Mo2—S2—Mo3	76.72 (3)	O2—Mo1—Mo3—S9	176.75 (10)
S2—Mo2—Mo1—Mo3	−64.18 (3)	O2—Mo1—Mo3—S10	14.31 (10)
S2—Mo2—Mo1—S1	169.67 (3)	O2—Mo1—Mo3—N1	94.58 (10)
S2—Mo2—Mo1—S2	−0.00 (3)	S1—Mo1—S2—Mo2	−8.80 (3)
S2—Mo2—Mo1—S4	−101.59 (3)	S1—Mo1—S2—Mo3	−86.11 (3)
S2—Mo2—Mo1—S7	9.48 (3)	S2—Mo1—S1—Mo2	8.86 (3)
S2—Mo2—Mo1—S8	158.04 (3)	S1—Mo1—S4—Mo3	95.93 (3)
S2—Mo2—Mo1—O2	82.25 (3)	S4—Mo1—S1—Mo2	−98.84 (3)
Mo1—Mo2—S3—Mo3	−42.75 (3)	S1—Mo1—S7—P2	24.12 (11)
S3—Mo2—Mo1—Mo3	38.49 (3)	S7—Mo1—S1—Mo2	144.77 (9)
S3—Mo2—Mo1—S1	−87.65 (3)	S1—Mo1—S8—P2	−166.51 (4)
S3—Mo2—Mo1—S2	102.67 (3)	S8—Mo1—S1—Mo2	172.04 (3)
S3—Mo2—Mo1—S4	1.08 (3)	S1—Mo1—O2—C1	−54.8 (2)
S3—Mo2—Mo1—S7	112.16 (4)	O2—Mo1—S1—Mo2	87.64 (9)
S3—Mo2—Mo1—S8	−99.28 (3)	S2—Mo1—S4—Mo3	−14.41 (3)
S3—Mo2—Mo1—O2	−175.07 (3)	S4—Mo1—S2—Mo2	91.62 (3)
Mo1—Mo2—S5—P1	−160.76 (2)	S4—Mo1—S2—Mo3	14.30 (3)
S5—Mo2—Mo1—Mo3	−76.12 (4)	S2—Mo1—S7—P2	162.70 (4)
S5—Mo2—Mo1—S1	157.73 (5)	S7—Mo1—S2—Mo2	−174.15 (3)
S5—Mo2—Mo1—S2	−11.94 (4)	S7—Mo1—S2—Mo3	108.53 (3)
S5—Mo2—Mo1—S4	−113.54 (4)	S2—Mo1—S8—P2	−39.45 (12)
S5—Mo2—Mo1—S7	−2.46 (5)	S8—Mo1—S2—Mo2	−132.21 (10)
S5—Mo2—Mo1—S8	146.10 (4)	S8—Mo1—S2—Mo3	150.47 (10)
S5—Mo2—Mo1—O2	70.31 (5)	S2—Mo1—O2—C1	54.5 (2)
Mo1—Mo2—S6—P1	160.39 (2)	O2—Mo1—S2—Mo2	−90.56 (7)
S6—Mo2—Mo1—Mo3	138.21 (4)	O2—Mo1—S2—Mo3	−167.87 (7)
S6—Mo2—Mo1—S1	12.06 (4)	S4—Mo1—S7—P2	−92.28 (4)
S6—Mo2—Mo1—S2	−157.61 (4)	S7—Mo1—S4—Mo3	−103.11 (4)
S6—Mo2—Mo1—S4	100.79 (4)	S4—Mo1—S8—P2	98.61 (4)
S6—Mo2—Mo1—S7	−148.13 (4)	S8—Mo1—S4—Mo3	179.39 (3)
S6—Mo2—Mo1—S8	0.43 (5)	S7—Mo1—S8—P2	3.69 (4)
S6—Mo2—Mo1—O2	−75.36 (4)	S8—Mo1—S7—P2	−3.67 (4)
Mo1—Mo2—O1—C1	2.5 (2)	S7—Mo1—O2—C1	143.1 (3)
O1—Mo2—Mo1—Mo3	−147.21 (8)	O2—Mo1—S7—P2	81.54 (9)
O1—Mo2—Mo1—S1	86.64 (8)	S8—Mo1—O2—C1	−138.8 (3)
O1—Mo2—Mo1—S2	−83.03 (8)	O2—Mo1—S8—P2	−80.75 (8)
O1—Mo2—Mo1—S4	175.38 (8)	Mo2—Mo3—S2—Mo2	−0.0
O1—Mo2—Mo1—S7	−73.55 (8)	Mo2—Mo3—S2—Mo1	74.22 (3)
O1—Mo2—Mo1—S8	75.01 (8)	Mo2—Mo3—S3—Mo2	−0.0
O1—Mo2—Mo1—O2	−0.77 (8)	Mo2—Mo3—S4—Mo1	−40.044 (19)
Mo3—Mo2—S1—Mo1	45.210 (18)	Mo2—Mo3—S9—P3	99.86 (5)
S1—Mo2—Mo3—Mo1	−41.29 (2)	Mo2—Mo3—S10—P3	−150.03 (3)
S1—Mo2—Mo3—S2	−107.64 (3)	Mo2—Mo3—N1—C15	135.42 (15)
S1—Mo2—Mo3—S3	88.35 (3)	Mo2—Mo3—N1—C16	−45.2 (3)
S1—Mo2—Mo3—S4	−4.07 (3)	Mo1—Mo3—S2—Mo2	−74.22 (3)
S1—Mo2—Mo3—S9	90.51 (3)	Mo1—Mo3—S2—Mo1	0.0
S1—Mo2—Mo3—S10	173.41 (2)	Mo1—Mo3—S3—Mo2	41.38 (3)
S1—Mo2—Mo3—N1	−99.95 (3)	Mo1—Mo3—S4—Mo1	0.0
Mo3—Mo2—S2—Mo1	−76.72 (3)	Mo1—Mo3—S9—P3	−159.56 (3)
Mo3—Mo2—S2—Mo3	−0.0	Mo1—Mo3—S10—P3	159.31 (3)
S2—Mo2—Mo3—Mo1	66.35 (3)	Mo1—Mo3—N1—C15	88.5 (2)
S2—Mo2—Mo3—S2	0.00 (3)	Mo1—Mo3—N1—C16	−92.0 (2)
S2—Mo2—Mo3—S3	−164.01 (4)	S2—Mo3—S3—Mo2	−13.34 (4)
S2—Mo2—Mo3—S4	103.57 (4)	S3—Mo3—S2—Mo2	13.23 (4)
S2—Mo2—Mo3—S9	−161.84 (4)	S3—Mo3—S2—Mo1	87.45 (4)
S2—Mo2—Mo3—S10	−78.95 (4)	S2—Mo3—S4—Mo1	14.53 (3)
S2—Mo2—Mo3—N1	7.69 (4)	S4—Mo3—S2—Mo2	−88.57 (3)
Mo3—Mo2—S3—Mo3	−0.0	S4—Mo3—S2—Mo1	−14.35 (3)
S3—Mo2—Mo3—Mo1	−129.64 (4)	S2—Mo3—S9—P3	−32.90 (11)
S3—Mo2—Mo3—S2	164.01 (4)	S9—Mo3—S2—Mo2	145.50 (9)
S3—Mo2—Mo3—S3	0.00 (4)	S9—Mo3—S2—Mo1	−140.28 (9)
S3—Mo2—Mo3—S4	−92.42 (4)	S2—Mo3—S10—P3	157.48 (4)
S3—Mo2—Mo3—S9	2.16 (4)	S10—Mo3—S2—Mo2	104.37 (3)
S3—Mo2—Mo3—S10	85.06 (4)	S10—Mo3—S2—Mo1	178.59 (3)
S3—Mo2—Mo3—N1	171.70 (4)	S2—Mo3—N1—C15	141.7 (3)
Mo3—Mo2—S5—P1	138.91 (3)	S2—Mo3—N1—C16	−38.9 (2)
S5—Mo2—Mo3—Mo1	138.50 (3)	N1—Mo3—S2—Mo2	−174.66 (9)
S5—Mo2—Mo3—S2	72.14 (3)	N1—Mo3—S2—Mo1	−100.43 (9)
S5—Mo2—Mo3—S3	−91.86 (3)	S3—Mo3—S4—Mo1	−93.68 (3)
S5—Mo2—Mo3—S4	175.71 (3)	S4—Mo3—S3—Mo2	95.04 (3)
S5—Mo2—Mo3—S9	−89.70 (4)	S3—Mo3—S9—P3	101.59 (4)
S5—Mo2—Mo3—S10	−6.80 (3)	S9—Mo3—S3—Mo2	−178.65 (4)
S5—Mo2—Mo3—N1	79.83 (3)	S3—Mo3—S10—P3	−97.10 (4)
Mo3—Mo2—S6—P1	−93.21 (5)	S10—Mo3—S3—Mo2	−100.72 (3)
S6—Mo2—Mo3—Mo1	−133.96 (5)	S4—Mo3—S9—P3	−161.54 (4)
S6—Mo2—Mo3—S2	159.68 (5)	S9—Mo3—S4—Mo1	178.40 (3)
S6—Mo2—Mo3—S3	−4.32 (5)	S4—Mo3—N1—C15	35.6 (2)
S6—Mo2—Mo3—S4	−96.75 (5)	S4—Mo3—N1—C16	−145.0 (2)
S6—Mo2—Mo3—S9	−2.16 (6)	N1—Mo3—S4—Mo1	96.53 (7)
S6—Mo2—Mo3—S10	80.74 (5)	S9—Mo3—S10—P3	−9.20 (3)
S6—Mo2—Mo3—N1	167.37 (5)	S10—Mo3—S9—P3	9.29 (3)
Mo3—Mo2—O1—C1	−37.4 (3)	S9—Mo3—N1—C15	−51.1 (2)
O1—Mo2—Mo3—Mo1	47.02 (11)	S9—Mo3—N1—C16	128.3 (3)
O1—Mo2—Mo3—S2	−19.33 (11)	N1—Mo3—S9—P3	−72.85 (9)
O1—Mo2—Mo3—S3	176.66 (11)	S10—Mo3—N1—C15	−130.2 (2)
O1—Mo2—Mo3—S4	84.24 (11)	S10—Mo3—N1—C16	49.2 (2)
O1—Mo2—Mo3—S9	178.83 (11)	N1—Mo3—S10—P3	74.30 (8)
O1—Mo2—Mo3—S10	−98.28 (11)	Mo2—S5—P1—S6	3.82 (5)
O1—Mo2—Mo3—N1	−11.64 (12)	Mo2—S5—P1—O3	129.05 (7)
S1—Mo2—S2—Mo1	8.82 (3)	Mo2—S5—P1—O4	−122.87 (6)
S1—Mo2—S2—Mo3	85.54 (4)	Mo2—S6—P1—S5	−3.77 (5)
S2—Mo2—S1—Mo1	−8.93 (3)	Mo2—S6—P1—O3	−128.29 (6)
S1—Mo2—S3—Mo3	−96.93 (3)	Mo2—S6—P1—O4	122.52 (7)
S3—Mo2—S1—Mo1	98.35 (3)	Mo1—S7—P2—S8	4.79 (5)
S1—Mo2—S5—P1	−41.69 (10)	Mo1—S7—P2—O5	−121.83 (6)
S5—Mo2—S1—Mo1	−134.19 (8)	Mo1—S7—P2—O6	130.45 (6)
S1—Mo2—S6—P1	170.16 (4)	Mo1—S8—P2—S7	−4.78 (5)
S6—Mo2—S1—Mo1	−172.13 (3)	Mo1—S8—P2—O5	120.71 (7)
S1—Mo2—O1—C1	57.0 (2)	Mo1—S8—P2—O6	−130.39 (6)
O1—Mo2—S1—Mo1	−87.04 (7)	Mo3—S9—P3—S10	−12.23 (5)
S2—Mo2—S3—Mo3	13.31 (4)	Mo3—S9—P3—O7	110.50 (5)
S3—Mo2—S2—Mo1	−89.92 (4)	Mo3—S9—P3—O8	−136.70 (6)
S3—Mo2—S2—Mo3	−13.19 (4)	Mo3—S10—P3—S9	12.29 (5)
S2—Mo2—S5—P1	−170.59 (4)	Mo3—S10—P3—O7	−108.55 (7)
S5—Mo2—S2—Mo1	172.05 (3)	Mo3—S10—P3—O8	137.56 (6)
S5—Mo2—S2—Mo3	−111.23 (4)	S5—P1—O3—C3	−60.3 (3)
S2—Mo2—S6—P1	37.79 (11)	S5—P1—O4—C5	69.4 (3)
S6—Mo2—S2—Mo1	137.81 (9)	S6—P1—O3—C3	61.1 (3)
S6—Mo2—S2—Mo3	−145.47 (9)	S6—P1—O4—C5	−53.4 (3)
S2—Mo2—O1—C1	−53.1 (2)	O3—P1—O4—C5	−172.8 (3)
O1—Mo2—S2—Mo1	88.99 (8)	O4—P1—O3—C3	−179.0 (3)
O1—Mo2—S2—Mo3	165.71 (9)	S7—P2—O5—C7	52.4 (3)
S3—Mo2—S5—P1	84.87 (4)	S7—P2—O6—C9	−66.6 (3)
S5—Mo2—S3—Mo3	99.49 (4)	S8—P2—O5—C7	−69.6 (3)
S3—Mo2—S6—P1	−96.67 (4)	S8—P2—O6—C9	55.2 (3)
S6—Mo2—S3—Mo3	177.38 (4)	O5—P2—O6—C9	175.4 (3)
S5—Mo2—S6—P1	2.92 (4)	O6—P2—O5—C7	171.0 (3)
S6—Mo2—S5—P1	−2.90 (4)	S9—P3—O7—C11	−171.99 (16)
S5—Mo2—O1—C1	−138.1 (3)	S9—P3—O8—C13	57.4 (3)
O1—Mo2—S5—P1	−89.05 (8)	S10—P3—O7—C11	−52.0 (3)
S6—Mo2—O1—C1	143.2 (3)	S10—P3—O8—C13	−64.9 (3)
O1—Mo2—S6—P1	86.06 (9)	O7—P3—O8—C13	174.8 (3)
Mo2—Mo1—Mo3—Mo2	0.0	O8—P3—O7—C11	67.9 (3)
Mo2—Mo1—Mo3—S2	65.706 (11)	Mo2—O1—C1—O2	−3.8 (6)
Mo2—Mo1—Mo3—S3	−38.384 (11)	Mo2—O1—C1—C2	173.5 (2)
Mo2—Mo1—Mo3—S4	−131.623 (14)	Mo1—O2—C1—O1	2.8 (6)
Mo2—Mo1—Mo3—S9	−134.09 (3)	Mo1—O2—C1—C2	−174.6 (2)
Mo2—Mo1—Mo3—S10	63.46 (2)	P1—O3—C3—C4A	−130.7 (4)
Mo2—Mo1—Mo3—N1	143.738 (14)	P1—O3—C3—C4B	163.3 (4)
Mo2—Mo1—S1—Mo2	0.0	P1—O4—C5—C6	−106.7 (4)
Mo2—Mo1—S2—Mo2	−0.0	P2—O5—C7—C8	100.0 (4)
Mo2—Mo1—S2—Mo3	−77.31 (2)	P2—O6—C9—C10	−158.5 (3)
Mo2—Mo1—S4—Mo3	41.55 (3)	P3—O7—C11—C12	−174.7 (2)
Mo2—Mo1—S7—P2	154.96 (3)	P3—O8—C13—C14	−122.1 (3)
Mo2—Mo1—S8—P2	−157.05 (2)	C25—O9—C27—C28	−170.2 (6)
Mo2—Mo1—O2—C1	−0.5 (2)	C27—O9—C25—C26	−176.0 (6)
Mo3—Mo1—S1—Mo2	−45.398 (19)	Mo3—N1—C15—C17	176.6 (3)
S1—Mo1—Mo3—Mo2	41.31 (3)	Mo3—N1—C16—C18	−177.8 (2)
S1—Mo1—Mo3—S2	107.01 (3)	C15—N1—C16—C18	1.6 (5)
S1—Mo1—Mo3—S3	2.92 (3)	C16—N1—C15—C17	−2.8 (6)
S1—Mo1—Mo3—S4	−90.32 (3)	C23—N2—C24—C22	0.1 (8)
S1—Mo1—Mo3—S9	−92.79 (3)	C24—N2—C23—C21	0.2 (9)
S1—Mo1—Mo3—S10	104.77 (3)	N1—C15—C17—C19	2.0 (6)
S1—Mo1—Mo3—N1	−174.95 (3)	N1—C16—C18—C19	0.4 (6)
Mo3—Mo1—S2—Mo2	77.31 (2)	C15—C17—C19—C18	0.1 (6)
Mo3—Mo1—S2—Mo3	0.0	C15—C17—C19—C20	−179.4 (4)
S2—Mo1—Mo3—Mo2	−65.71 (3)	C16—C18—C19—C17	−1.2 (6)
S2—Mo1—Mo3—S2	0.00 (3)	C16—C18—C19—C20	178.3 (4)
S2—Mo1—Mo3—S3	−104.09 (3)	C17—C19—C20—C21	36.6 (6)
S2—Mo1—Mo3—S4	162.67 (3)	C17—C19—C20—C22	−143.4 (4)
S2—Mo1—Mo3—S9	160.20 (4)	C18—C19—C20—C21	−142.9 (4)
S2—Mo1—Mo3—S10	−2.24 (3)	C18—C19—C20—C22	37.1 (6)
S2—Mo1—Mo3—N1	78.03 (3)	C19—C20—C21—C23	−179.4 (4)
Mo3—Mo1—S4—Mo3	0.0	C19—C20—C22—C24	179.6 (4)
S4—Mo1—Mo3—Mo2	131.62 (3)	C21—C20—C22—C24	−0.3 (7)
S4—Mo1—Mo3—S2	−162.67 (3)	C22—C20—C21—C23	0.6 (7)
S4—Mo1—Mo3—S3	93.24 (3)	C20—C21—C23—N2	−0.6 (9)
S4—Mo1—Mo3—S4	0.00 (3)	C20—C22—C24—N2	−0.0 (8)
S4—Mo1—Mo3—S9	−2.47 (3)		

Symmetry codes: (i) −*x*, −*y*, −*z*; (ii) −*x*+1, −*y*, −*z*+1; (iii) −*x*, *y*−1/2, −*z*+1/2; (iv) −*x*, −*y*, −*z*+1; (v) *x*+1, *y*, *z*; (vi) −*x*, *y*+1/2, −*z*+1/2; (vii) *x*−1, *y*, *z*; (viii) *x*, −*y*+1/2, *z*+1/2; (ix) −*x*+1, *y*−1/2, −*z*+3/2; (x) *x*, −*y*+1/2, *z*−1/2; (xi) −*x*+1, *y*+1/2, −*z*+3/2; (xii) *x*, *y*, *z*+1; (xiii) −*x*−1, −*y*, −*z*; (xiv) *x*, *y*, *z*−1; (xv) *x*−1, −*y*+1/2, *z*−1/2; (xvi) *x*−1, *y*, *z*−1; (xvii) *x*+1, *y*, *z*+1; (xviii) −*x*+1, *y*+1/2, −*z*+1/2; (xix) *x*+1, −*y*+1/2, *z*+1/2; (xx) −*x*+1, *y*−1/2, −*z*+1/2.

### Hydrogen-bond geometry (Å, º)

**Table:**

*D*—H···*A*	*D*—H	H···*A*	*D*···*A*	*D*—H···*A*
C11—H11*B*···O5^vii^	0.99	2.38	3.335 (7)	161

Symmetry code: (vii) *x*−1, *y*, *z*.
